# Genotypic and Phenotypic Composition of Sickle Cell Disease in the Arab Population - A Systematic Review

**DOI:** 10.2147/PGPM.S391394

**Published:** 2023-02-21

**Authors:** Fateen Ata, Alaa Rahhal, Lujain Malkawi, Phool Iqbal, Ibrahim Khamees, Mousa Alhiyari, Zohaib Yousaf, Hana Qasim, Awni Alshurafa, Sundus Sardar, Saad Javed, Liam Fernyhough, Mohamed Yassin

**Affiliations:** 1 Department of Endocrinology, Hamad General Hospital, Hamad Medical Corporation, Doha, Qatar; 2 Department of Clinical Pharmacy, Hamad Medical Corporation, Doha, Qatar; 3 Department of Internal Medicine, Faculty of Medicine, Jordan University of Science and Technology, Irbid, Jordan; 4 Department of Internal Medicine, Metropolitan Hospital, New York, NY, USA; 5 Department of Internal Medicine, Hamad General Hospital, Hamad Medical Corporation, Doha, Qatar; 6 Department of Internal Medicine, Reading Hospital - Tower Health, West Reading, PA, USA; 7 Department of Internal Medicine, UMKC School of medicine, Kansas, MO, USA; 8 Department of Medical Oncology /Hematology, National Centre for Cancer Care and Research, Hamad Medical Corporation, Doha, Qatar; 9 Department of Medicine, Division of Nephrology, Pennsylvania State University College of Medicine, Hershey Medical Center, Hershey, PA, USA; 10 Department of Internal Medicine, Icahn school of medicine at Mount Sinai/Queens Hospital Center, New York, NY, USA; 11 Department of Medical Education, Weill Cornell Medicine – Qatar, Doha, Qatar

**Keywords:** sickle cell disease, genotypes, SCD, sickle cell anemia, Arab

## Abstract

Sickle cell disease (SCD) is a genetic disease influenced by ethnicity and regional differences in its clinical course. Recent advances in the management of SCD with newer therapies are being introduced to the Western population. However, many of these treatments are yet to be used in the Arabic SCD population. Understanding the genetic variations of SCD regionally is essential to anticipate the utilization of new treatments. This systematic review’s main objective is to pool the available data on the genetic composition of SCD in the Arabic population. Data for 44,034 patients was extracted from 184 studies (11 case reports, 8 case series, 56 retrospectives, 107 prospective observational studies, and 2 clinical trials) using PubMed, Scopus, and Google Scholar. Male (49%) and female (51%) patients were equally reported wherever gender was available (N=13105). Various SCD genotypes were reported in a total of 14,257 patients, including Hb SS (77%) Hb Sβ0 (9.9%), and Hb Sβ+ (7.2%), while the rest of the genotypes, including HbSC, HbSD, HbSE, HbSO Arab, Hb S/α-Thal, Hb Sβ0 + α-Thal, and HBS Oman were individually reported in <4% of the cases. Major SCD complications in the Arab population included pain crises (48.25%) followed by neurological complications (33.46%), hepatobiliary complications (25.53%), musculoskeletal complications (24.73%), and hemolytic anemia (23.57%). The treatments reported for SCD included hydroxyurea (20%), blood transfusion (14.32%), and Deferasirox (3.03%). We did not find the use of stem cell transplantation or newer treatments such as L-Glutamine, Voxelotor, Crizanlizumab, or gene therapy reported in any of the studies included in our review. This review highlights the genetic makeup of SCD in Arab countries and its common phenotypic manifestations and will help direct further research on SCD in this region, especially concerning genetic therapy.

**Systematic Review Registration:**

The protocol has been registered in the International Prospective Register of Systematic Reviews(PROSPERO):CRD42020218,666. https://www.crd.york.ac.uk/PROSPERO/display_record.php?RecordID=218666.

## Introduction

The first description of sickle cell anemia (SCA) like disorder was provided by Dr. Africanus Horton in his book “The disease of Tropical Climates and their treatment (1872). However, in 1910, Dr. James B Herrick and Dr. Ernest Irons reported sickle-shaped red blood cells in a dental student.^1^ Sickle cell disease (SCD) refers to various groups of hemoglobinopathies characterized by different autosomal recessive genetic mutations in the hemoglobin beta-subunit.^1–3^ As a consequence of these genetic mutations, deformed red blood cells (RBCs) are produced that are not suitable for the optimal supply of oxygen to the tissues.^2^ The RBCs become sickled and rigid and are unable to pass through small vascular channels, resulting in hemolysis and intravascular RBC clumping, causing tissue hypoxia, ischemia, infarction, and necrosis.^2^ There are potentially severe complications that carry high morbidity and mortality, such as delayed growth,^4^,^5^ delayed sexual maturation,^6^ acute chest syndrome, acute stroke,^7^ chronic kidney disease,^8^ splenic infarction, pulmonary artery hypertension, leg ulcers, myocardial infarction, and other body organ damage.^3^,^9–11^

Within the umbrella of SCD, subgroups exist such as sickle cell anemia (HbSS), hemoglobin SC disease (HbSC), and other combined mutations including hemoglobin sickle-beta-thalassemia (beta-thalassemia positive or beta-thalassemia negative).^1–3^ The highest prevalence of SCD is among the people of Sub-Saharan Africa, South Asia, the Middle East, and the Mediterranean.^12^ The incidence is estimated to be between 300,000 to 400,000 neonates globally each year.^3^ In a meta-analysis of the prevalence and associated mortality in children under five years of age, Wastnedge et al reported a global birth prevalence of homozygous sickle cell disease of 112 per 100,000 live births but 1125/100,000 in Africa compared to 43.12/100,000 in Europe.^13^ It has been estimated that the global burden of SCD will increase by up to 30% by 2050.^14^

The management of SCD includes universal newborn screening programs for early diagnosis and intervention.^15^,^16^ Current medical management includes hydroxyurea, which has been proven to reduce sickling of RBCs and thereby provide mortality benefits,^17^,^18^ L-glutamine for the prevention of acute pain episodes of SCD in patients five years of age or older,^19^ and more recently crizanlizumab and voxelotor.^17^,^20^ Additional supportive management includes blood transfusion, pain management, and avoidance of triggering factors.^17^ There is no current cure for the disease in adults, but bone marrow transplantation in children has been shown to be curative in selected patients.^15^ Gene therapy is still in the clinical trial stage but is very promising.^17^,^19^ In anticipation of the future implementation of gene therapy for SCD and to improve SCD care overall, it is important to understand the genotypic and phenotypic SCD landscape in different regions of the world. Although many studies have reported genotypic and phenotypic variants of SCD in specific countries, the last updated review in Arab world countries was published in 2011 by El-Hazmi eta. more than a decade ago and before newer interventions and testing were so widely available.^21^ With this systematic review, we aim to report the various genotypes, phenotypes, mutational variations, haplotypes, and associations between phenotypes and treatments in SCD in 22 Arab countries through a rigorous search of multiple research databases and thus contribute to the current literature for better future perspectives in SCD management.

## Materials and Methods

### Literature Search

A systematic literature search was performed for any date up to 25 November 2020 via electronic databases (PubMed, Scopus, and Google Scholar) to identify English-language articles relevant to the research question. The protocol has been described in detail and published, including the search terms and methodology.^22^ The following search terms were used in the literature review: “Genotype” OR “Genetics” OR “Gene” OR “Mutations” OR “Haplotype” AND “Sickle Cell Disease” OR “SCD” OR “Hemoglobin S/O” OR “Sickle cell anemia” OR “SCA” OR “sickle/ beta‐thalassemia”, “SC/SD” OR “Hb SS” OR “Hb SC” OR “Hb Sβ+” OR “Hb Sβ0” OR “HbSD” OR “HbSE” OR “HbSO Arab” OR “HBS Oman” AND “Arab” OR “Arab countries” OR “Arab Population” OR “The Middle East” OR “Algeria” OR “Bahrain” “Comoros” OR “Djibouti” OR “Egypt” OR “Iraq” OR “Jordan” OR “Kuwait” OR “Lebanon” OR “Libya” OR “Mauritania” OR “Morocco” OR “Oman” OR “Palestine” OR “Qatar” OR “Saudi Arabia” OR “Somalia” OR “Sudan” OR “Syria” OR “Tunisia” OR “the United Arab Emirates” OR “Yemen”.

Our pre-defined research question was to compile genotypes and phenotypes of all patients (pediatric and adult) with a confirmed diagnosis of SCD who had genotypes mentioned in the included studies from Arabic countries.

### Study Selection

#### Inclusion Criteria

All English language studies from Arabic countries describing original data on patients with confirmed SCD with genotypes mentioned were included in the review. The Arab world is defined by 22 Arab countries which are members of the Arab league.^23^

#### Exclusion Criteria

Studies reporting secondary (already published) data were excluded from this review. Articles in languages other than English were also excluded from the review. Studies where original data of SCD patients was reported but genotypes were not mentioned were also excluded.

Two reviewers independently screened the finalized studies via title, abstract, and keywords. Shortlisted studies then underwent a detailed full-length review. Disagreements in the screening process were resolved by an independent review from a third member.

### Bias Assessment

The Joanna Briggs Institute case report appraisal checklist for inclusion in systematic reviews and the Methodological index for non-randomized studies (MINORS) assessment tools were used for quality assessment for case reports and larger observational studies, respectively.^24^,^25^

### Data Collection and Statistical Analysis

Data collected include socio-demographic variables, genetic composition, phenotypic manifestations, and various managements of SCD patients, subject to availability. Descriptive and summary statistics were used with data presented as mean (with standard deviations), median (with interquartile ranges) and numbers (with percentages) as appropriate. Data was collected by 4 reviewers independently with cross checking of data by 2 reviewers.

Preferred Reporting Items for Systematic Reviews and Meta-Analyses (PRISMA) guidelines were followed for the synthesis of this systematic review.^26^

## Results

**Figure 1:**
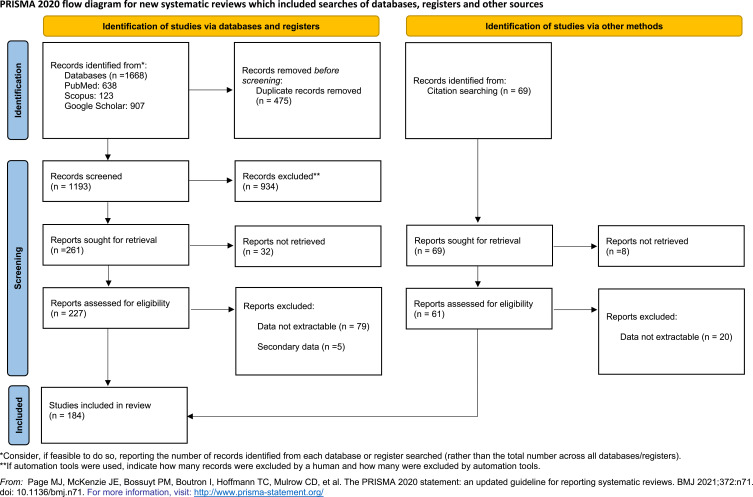
PRISMA flow diagram of the article screening process with the details of included and excluded studies.

184 eligible studies reported 44,034 SCD patients in the Arabic population (Figure 1). The studies included 11 case reports, 8 case series, 56 retrospective, 107 prospective observational studies, and 2 clinical trials (one randomized and one nonrandomized) (Supplementary File 1).

As shown in Table 1, male gender represented 49% and female gender represented 51% of cases (given that the gender data was only available for 13,105 patients). More than half of the cases were reported from Saudi Arabia, followed by Bahrain with 27.24% of the cases. Fewer than 5% of the cases each were reported from Jordan, Egypt, Kuwait, and Tunisia, and less than 2% each from the remaining (Table 1). Saudi Arabia was also the leading country in the Arab region in terms of the number of SCD studies with genotype information published, followed by Kuwait then Egypt (Table 2).

**Table 1:** Baseline Characteristics of the Published Data of Patients with SCD in the Arab Population (N= 44,034)

Characteristic	N (%)
**Gender** Total	N=13105
Male	6415 (49)
Female	6690 (51)
**Country**	
Algeria	84 (0.19)
Bahrain	12,209 (27.5)
Egypt	1550 (3.5)
Iran	28 (0.06)
Iraq	415 (0.9)
Jordan	2082 (4.7)
Kuwait	1520 (3.4)
Lebanon	453 (1)
Libya	5 (0.01)
Morocco	124 (0.2)
Oman	836 (1.8)
Palestine	151 (0.3)
Qatar	2 (0.004)
Saudi Arabia	22,708 (51.3)
Sudan	476 (1)
Tunisia	1104 (2.5)
UAE	147 (0.3)
Yemen	256 (0.5)
**Genotypes**	
Genotypes reported	14,257 (32.3)
HbSS	10980 (77)
HbSβ^0^	1415 (9.9)
HbSβ^+^	1037 (7.2)
HbSC	502 (3.5)
HbS/α-Thal	339 (2.3)
HBSD	66 (0.4)
HbSO-Arab	56 (0.3)
Hb Sβ^0^ + Alpha Thal	42 (0.3)
HBSE	16 (0.1)
HBS-Oman	1 (0.007)

**Table 2:** Number of Studies Published per Country (N=184)

Country	No. of Studies	Percentage
Saudi Arabia	61	33.2
Kuwait	21	11.4
Egypt	20	10.9
Oman	15	82
Tunisia	15	82
Bahrain	14	7.6
Sudan	7	3.8
Jordan	6	3.3
Lebanon	6	3.3
Yemen	5	2.7
Morocco	3	1.6
Iran	2	1.1
Iraq	2	1.1
Palestine	2	1.1
UAE	2	1.1
Algeria	1	0.5
Libya	1	0.5
Qatar	1	0.5

Genotypes were available for 14,257 of the 44,034 cases (32.7%). The most common SCD genotype reported in this population was Hb SS, present in 77% of cases, followed by Hb Sβ^0^ in 9.9% and Hb Sβ^+^ in 7.2%, and HbSC in 3.5%, while the rest of the genotypes, including HbS/α-Thal, HbSD, HbSE, HbSO-Arab, HbSβ^0^ + Alpha Thal, and HBS-Oman were individually reported in <4% of the cases (N=520). (Table 1).

The most commonly reported complication of SCD was pain crisis (48.25%), followed by neurological complications (33.46%), hepatobiliary complications (25.53%), musculoskeletal complications (24.73%), and hemolytic anemia (23.57%) (Table 3). Other complications were individually reported in <15% of cases. Additionally, as shown in Table 4, treatment for SCD included hydroxyurea in around 20% of the subjects, blood transfusion in 14.32%, and Deferasirox in 3.03%. Not all studies included complication and management data.

**Table 3:** Complications of Patients with SCD in the Arab Population (N= 44,034)

Complication	Frequency	Population*	Percentage
Pain crisis	1161	2407	48.23
Neurological complications	1526	4560	33.46
Hepatobiliary complications	1087	4258	25.53
Musculoskeletal complications	1277	5163	24.73
Hemolytic anemia	434	1841	23.57
Pulmonary complications	565	3857	14.65
Acute splenic sequestration	335	2826	11.85
Renal complications	163	2117	7.70
Functional asplenia	85	1498	5.67
Hyper viscosity	77	1430	5.38
Transfusion hemosiderosis	50	1920	2.60
Ophthalmologic complications	76	3329	2.28
Dermatological complications	10	1359	0.74

**Notes**: *Data readily available from the published studies included in the review as the complications were not reported in all the studies identified. Reported from highest to lowest frequency.

**Table 4:** Treatment Options Used for Patients with SCD in the Arab Population (N= 44,034)

Treatment	Frequency	Population*	Percentage
Blood transfusion	1022	7144	14.3
Deferasirox	30	989	3.03
Hydroxyurea	663	3332	19.9
Stem cell transplantation	0		
L-Glutamine			
Voxelotor			
Crizanlizumab			
Gene therapy			

**Note**: *Data readily available from the published studies included in the review as complications were not reported in all the studies identified.

## Discussion

This study represents a systematic and comprehensive review of all the prior published peer-reviewed articles describing genotypes of SCD in the Arabic population. We captured 44,034 patients diagnosed with SCD in all Arab countries up to November 2020 (Table 1). The Arab world covers over 13 million km^2^ from North Africa to the Middle East, with a population of over 400 million.^23^ Arab populations exhibit high levels of genetic diversity which is mainly due to the frequent historical migrations of Arab tribes that lead to an admixture of Arabs with other populations from Asia, Europe, and Africa. Additionally, the high rate of consanguineous marriage and the large size of Arab families has contributed to a high prevalence of autosomal recessive diseases in the area, including SCD.^27^ Variability in genetic disease manifestations and prognosis based on ethnic differences is well-known.^28^ As the era of genomic medicine unfolds, it becomes imperative to delineate and understand such variations with a view to furthering personalized medicine in the clinical setting.^29^ As Arab ethnicity is considerably different from Asian or Western ethnic groups, genetic data from the Arab world needs to be compiled separately for a more accurate genomic assessment.

SCD consists of a group of hemoglobinopathies, all of which contain at least one hemoglobin S allele (HbS) and another abnormal beta-hemoglobin gene (HBB), resulting in disordered hemoglobin polymerization. Two HbS genes (HbSS) is most common and accounts for 60–70% of the SCD cases in the US.^30^ Other pathogenic HBB variants can be combined with HbS to cause SCD such as HbC, HbE, Hbβ^0^, Hbβ^+^, HbD, and HbO Arab.^31^ In our review, the genotype was available for 14,482 patients which is around 32.7% of all patients in the studies. HbSS was the most common genotype reported in the Arab population at 77% of those reported, followed by HbSβ^0^ (9.9%) and HbSβ^+^ (7.2%) (Table 1). These show higher percentages of HbSS and HbSβthal and lower frequencies of HbSC than reported in the US in 2009, where SCD genotypes showed an HbSS frequency of 60%, HbSC (30%) and HbSβthal (10%). This was attributed to the large number of African American and Hispanic people in the US.^30^

Of interest, 16 patients had the HbSE genotype constituting around one-quarter of the cases of HbSE disease reported worldwide.^32^ Although HbSE has been considered to have a benign course, recent reviews have shown that HbSE can manifest with severe signs and symptoms of sickling, including acute chest syndrome. Mortality related to severe manifestations of HbSE has been reported in at least 3 cases. These patients died due to ischemia with severe sickle cell crisis, cardiopulmonary collapse secondary to sickle crisis, and massive marrow embolism during admission with pain crisis.^33–35^

It is important to note that some genotypes such as HbSO-Arab and HBS Oman are primarily seen in Arab ethnicities. Our review found HbSO-Arab and HBS Oman in 56 cases and one case respectively.^36–43^ Although their prevalence is low, attention is required as genetic therapy might differ for these genotypic variations if a curative treatment is intended.

Saudi Arabia contributed the highest number of SCD cases in our review (Table 1). This has previously been attributed to the prevalence of consanguineous marriages of up to 57.7% of all marriages.^44^ Several premarital screening programs and awareness programs have been instituted to decrease the prevalence of SCD in Saudi Arabia. These have been successful in decreasing the number of at-risk marriages, but the limited access to health services, the cultural stigmas, and the religious beliefs have made effective genetic counseling and screening difficult.^45^

Vaso-occlusive pain crisis has been reported previously as the main complication associated with SCD and the primary cause of hospitalization in SCD patients.^46^ Similarly in our review, vaso-occlusive pain or pain crisis was the most common reported complication (48.23%) (Table 3). This could potentially reflect different phenotypes in the Arab world. However, underreporting of complications and unpublished data could have had an impact on our results.

SCD causes several neurologic complications, such as large artery intracranial occlusive disease, thought to arise due to repeated sickling episodes causing endothelial hyperplasia and intraluminal thrombosis. It can also cause ischemic and hemorrhagic stroke, posterior cerebral encephalopathy syndrome, and cerebral fat embolism.^47^ In our review, 1526 patients were reported with one or more neurologic complications, indicating a high frequency in SCD patients in the Arab population (Table 3). However, neurologic complications of SCD may be rising globally and the contribution of ethnicity would require a comparative analysis of large studies of different ethnicities. Regardless, neurologic sequela of SCD can have significant impacts on quality of life in children and there have been recommendations for screening children who have poor academic performance via MRI imaging to rule out silent brain infarction (SBI) secondary to SCD.^47^ Similarly, transcranial Doppler ultrasound (TCD) has also been suggested to screen for new neurological events in cases with or without silent cerebral infarctions.^48^ This suggestion comes from a prospective cohort study on 421 children with SCD. The incidence of a new cerebral event in patients who had TCD with SBI in the background was higher (1.71 per 100 patient-years) compared to those who had TCD with no SBI (0.47 per 100 patient-years).^48^ Hence, TCD can be a valuable tool in neurologic risk stratification in patients with SCD.

Anemia and vaso-occlusion in SCD can lead to ischemia and end-organ damage, including musculoskeletal complications such as bone infarction, osteoporosis, and osteonecrosis.^49^ In our review, 1277 SCD patients were reported to have musculoskeletal complications (Table 3). Vaso-occlusion can also lead to pulmonary complications like acute chest syndrome, chronic pulmonary hypertension, and airway hyper-responsiveness.^50^,^51^ In our review, 565 SCD patients were reported to suffer from such complications (Table 3).

Sickle cells can aggregate in the spleen and can result in acute splenic sequestration crises (ASSC), a serious complication in young SCD patients.^52^ We found 335 Arab world SCD patients (11.85%) with this complication (Table 3), more frequent than reported in Africa, possibly because of the older age of the patients reported in the African studies.^53^ The studies from the African region have reported ASSC varying from 2% to 27.3%, with an overall prevalence of <10%. The single study that reported a significantly higher percentage (27.3%) compared to other added studies did not document specific details of ASSC.^54^ SCD patients can also suffer from hepatobiliary complications, including gallstones due to hyperbilirubinemia induced by hemolysis.^55^ We found 25% of the patients reported having SCD related hepatobiliary complications (Table 3), less than reported in one study from France where 40% of the children with SCD were suffering from hepatobiliary complications.^56^ Although this difference might be due to the different age groups in both studies (the French study included only patients <18 years of age whereas our review reports ages ranging from 1 years to 74 years), demographic variations in the patient population might also influence the phenotypic manifestations of these genotypes of SCD. Larger studies on genotypic and phenotypic correlations of SCD variants from the Arab world would serve to better answer this and validate our results.

Hydroxyurea has been approved by the FDA for the reduction of painful crises and the need for blood transfusion in homozygous SCD patients.^57^ It does so by increasing HbF levels, decreasing the proportion of the mutated Hb and resulting in less sickling.^58^ In our review, only 19.9% of the population for whom hydroxyurea use was reported took hydroxyurea (Table 4). This is low compared to the percentage reported in the USA which showed that one-third of the population included in that study had at least one hydroxyurea prescription.^59^ This might be attributed to the lower level of healthcare facilities and limited access to mediations in some Arab countries but it also represents an opportunity for improvement in the Arab world.

Red cell transfusion is an essential management component for SCD patients, provided as simple or exchange transfusions. There are multiple indications for red cell transfusion in SCD patients.^60^ However, chronic transfusion can cause iron overload, particularly in the liver and heart, for which iron chelation therapy becomes necessary.^61^ Only 14.3% of our study population for whom transfusion therapy was reported had received a blood transfusion, and only 3% of patients received deferasirox to treat iron overload (Table 4). This transfusion rate is much lower than the percentages reported in some African countries like Senegal (28.5%),^62^ and Congo (80.6%).^63^ Additionally, the reported use of deferasirox is low. However, it is known that chelation therapy has a low adherence rate. In the RELATH study (Registry of Latin Americans with Transfusional Hemosiderosis), around 46% of patients were reported to receive chelation therapy, out of which 20% were not compliant.^64^ Another potential reason is the cost of deferasirox, which is estimated to be as high as £26,061 (30 mg/kg dose) per annum for an average 70kg adult.^65^ Although the reason for comparatively low chelation rates in Arab countries is not apparent, it could be due to the fact that the articles included in this manuscript mainly focused on genotypes of SCD rather than its management. It would seem imperative to further explore treatment rate variations based on regional differences. Some possible reasons could include the cost of treatment, availability of treatment in various regions of the Arab world, access to healthcare, and education about the disease and its management in the general population.

The only well-established curative therapy for SCD patients currently is hematopoietic stem cell transplantation.^66^ Among the patient population of our review (patients who had genotypes specified), none of them reported stem cell transplantation. Newer therapies have been approved or are being studied in the management of SCD, among which gene therapy has great potential. Although there are several challenges ahead, with the rapid progress and significant research efforts in gene therapy, it is expected that it will help not only SCD but all hemoglobinopathies in the near future.^67^ Voxelotor is an HbS polymerization inhibitor that’s been approved by the FDA to treat SCD in patients 12 years or older. Patients taking Voxelotor 1500 mg were shown to have a rapid improvement in Hb concentration and a potential reduction in morbidity related to hemolytic anemia in SCD 72 weeks after starting the drug, without significant side effects compared to placebo.^68^ L-glutamine has also been approved by the FDA to treat SCD. It has been shown to decrease the frequency of pain crises in SCD patients aged 5 years and older over 48 weeks.^69^ More recently the FDA has approved Crizanlizumab in the treatment of SCD. This is a monoclonal antibody that binds to P-selectin on platelets and endothelial cells, helping to decrease the frequency of vaso-occlusive crises. In SCD patients aged 16 years and older it has shown a significant reduction in the frequency of vaso-occlusive crises.^70^ In our search, we did not find reports of any patients in the Arab countries who were treated with these newer drugs. Multiple trials are ongoing for the new therapies in SCD, such as Glutamine (NCT05371184^71^), Crizanlizumab (NCT03814746,^72^ NCT03474965,^73^ NCT04657822^74^), and Voxelotor (NCT03573882,^75^ NCT04188509,^76^ NCT02850406,^77^ NCT03036813,^78^ NCT04218084^79^) in SCD in the Arab countries, including Egypt, Oman, Lebanon, Saudi Arabia, and Jordan. As these newer therapies are introduced and reported on in the Arab world, we look forward to better evidence for the efficacy of these treatments in different patient populations.

Our review has limitations, some of which are inherent to the study design used. Firstly, we could only extract and analyze the readily available data from each study, which may have resulted in missing data. Secondly, the true prevalence of various genotypes and phenotypes of SCD might be different than reported as a result of unpublished and unreported data. Thirdly, we could not find any randomized controlled trials to strengthen our findings. We also note that there were no available data for four countries in the Arab world and that more than 75% of the cases reported were from Saudi Arabia and Bahrain, potentially limiting the representation and applicability across the other included countries. Also, other studies reporting data on complications and treatment may have been available but not included in this review if they did not also include genotype data. We also note that only English language articles were included despite the research population being Arabic speaking, potentially causing under-representation from countries that tend to publish in languages other than English. Nevertheless, this review represents the most updated and extensive data on genotypes and phenotypes of SCD across the Arabic world, laying a foundation for future research on the topic, especially concerning gene therapy in SCD.

## Conclusion

SCD is a common hemoglobinopathy in the Arab population. Data with regards to its genotypic makeup in the region is scattered and is different from other parts of the world. Many of the approved treatment modalities are yet to be reported in this patient population. Our review highlights the genetic makeup of SCD in the Arab countries with the common phenotypic manifestations. This data will help the direction of further research on SCD in this region, especially with respect to the upcoming era of genetic therapy.
